# The role of new echocardiographic techniques in athlete’s heart

**DOI:** 10.12688/f1000research.6745.1

**Published:** 2015-07-20

**Authors:** Antonello D'Andrea, Eduardo Bossone, Juri Radmilovic, Pio Caso, Raffaele Calabrò, Maria Giovanna Russo, Maurizio Galderisi

**Affiliations:** 1 Second University of Naples, Monaldi Hospital, Caserta, CE, 81100, Italy; 2 Cardiology Division, Fisciano, SA, 84084, Italy; 3 Department of Advanced Biomedical Sciences, Federico II University Hospital, Naples, 80138, Italy

**Keywords:** athlete’s heart, left ventricular hypertrophy, strain, tissue Doppler, three-dimensional echocardiography, sport

## Abstract

‘Athlete’s heart’ is a common term for the various adaptive changes induced by intensive exercise. Exercise causes alterations of the heart in hemodynamic response to the increased systemic and pulmonary demand during exercise. The understanding of these adaptations is of high importance, since they may overlap with those caused by pathological conditions. Cardiac imaging assessment of the athlete’s heart should begin with a complete echocardiographic examination. In recent years classical echocardiographic surveys have been joined by new developments: tissue Doppler imaging, strain rate echocardiography, and real-time 3-dimensional echocardiography. This review paper focuses on the importance of these new echocardiographic techniques in delineating the morphological characteristics and functional properties of the athlete’s heart.

## The athlete’s left heart

### Standard echocardiographic analysis

Long term physical training causes structural, functional and electrical changes in the heart that are physiological responses to the hemodynamic demands of increased cardiac output during effort. This adaptive remodelling can be defined as “athlete’s heart”.

The understanding of these changes is of high importance, since they have to be distinguished from those caused by pathological conditions. Moreover, there is some evolving evidence suggesting that some of the exercise-induced changes may be associated with acute and chronic cardiac damage and that in a small number of athletes this may predispose to atrial and ventricular arrhythmias. Thus, the need for a standardization of cardiovascular pre-participation screening of competitive athletes for sports eligibility has emerged, since athletes with underlying, masked cardiomyopathy may be at risk of lethal consequences during physical exertion ^1, 2^.

According to the Morganroth’s original hypothesis, two main models of training can be identified, which cause two distinct patterns of cardiac remodelling (myocardial hypertrophy) ^2^. Endurance training characterizes aerobic sports with dynamic-isotonic muscular involvement – such as long-distance swimming and running. These activities cause a gradual decrease in systemic arterial resistance and an increase in venous return, with a predominant volume overload, with higher left ventricular (LV) end diastolic volume (EDV) and stroke volume (eccentric hypertrophy).

On the other hand, strength training is typical of anaerobic sports characterized by predominant static-isometric muscular exercise, such as body-building, short-distance running and swimming. These sports categories cause mainly an increase in myocardial wall thickness rather than cavity diameters (concentric remodelling and hypertrophy), in response to the predominant pressure overload.

Morganroth’s original hypothesis has been criticized, because cardiac remodelling is also influenced by other factors like ethnicity, age, sex, genetics and body size. Moreover it has to be noted that most sports are actually characterized by a variable combination of both endurance and strength exercise, rather than only one of them.

Standard echocardiography has an essential role in assessing the characteristics of the athlete’s heart and in differentiating physiological and pathological LV hypertrophy (LVH) ^3^. Previous authors ^4^ in a large series of top level athletes reported that 55% had increased LV end-diastolic diameter and only 15% of them had values > 60 mm, even if ejection fraction (EF) was normal. Competitive athletes have LVH, involving all myocardial segments, with a maximal septal thickness < 12 mm. Conversely, patients with hypertrophic cardiomyopathy (HCM) show increased wall thickness (>15 mm), mainly in the basal septum, and in 20% of cases there is systolic anterior motion (SAM) of the mitral valve, or aortic valve mid-systolic closure ^5^. After a deconditioning period of at least three months a reduction in wall thickness can be observed in athletes, but not in HCM.

Identification of HCM is challenging, when wall thickness is between 13 and 15 mm (the so-called grey-zone of LVH) ^7^. In the last few years, development of new echocardiographic techniques have improved the knowledge of the athlete’s heart and differential diagnosis of physiological and pathological LVH.

### New left ventricular echocardiographic techniques

In the athlete’s heart, LV diastolic function is often supranormal, in particular in endurance-trained athletes, when compared with untrained individuals. LV remodelling in athletes is associated with normal or increased myocardial relaxation, as an expression of increased elastic recoil, different from HCM patients, in whom diastolic dysfunction may be the first expression of the disease and may precede the development of LVH ^8^.

In athletes transmitral E/A ratio is often > 2, with typical low A velocity (late diastole), and this parameter is useful to distinguish this condition from pathological LVH, where E/A ratio is < 1 and E velocity deceleration time is prolonged ^9^.

**Figure 1.**
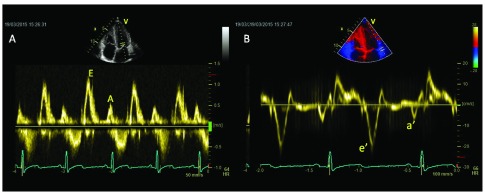
Transmitral flow pattern (left panel, 1a) and tissue Doppler (right panel, 1b) of an endurance athlete, showing supranormal diastolic function both at a global and regional level.

Pulsed tissue Doppler (TDI)-derived early diastolic myocardial velocity (e’) of basal septal and basal lateral wall is increased in athletes. Conversely, HCM is characterised by an e’ reduction in both the hypertrophic septum and the normal thickness of lateral wall ^10^. Lewis *et al.* suggested that an e’ peak velocity threshold of < 11.5 cm/sec on TDI can be useful to raise suspicion for pathological LVH ^11^ ( Figure 1).

Athletes have no regional diastolic dysfunction (e’/a’ < 1), while this is evident in 25% of myocardial segments of HCM patients and in hypertensive patients ^12^.

Finally E/e’ ratio is low in athletes, but increased in HCM patients, it being related with NYHA class and exercise capacity. Reduction of e’ velocity of both septal and lateral annulus is common after ultra-long duration exercises ^13^.

Moreover, pulsed TDI gives additional information regarding myocardial systolic performance at rest, showing normal or supranormal values in athlete’s heart ^14^. In athletes, LVH is combined with normal EF, normal or supranormal stroke volume and systolic peak velocity (s’) > 9 cm/sec, while in pathological LVH (HCM or arterial hypertension) s’ is < 9 cm/sec, with EF normal or high in early stages and reduced in advanced stages ^15^.

**Figure 2.**
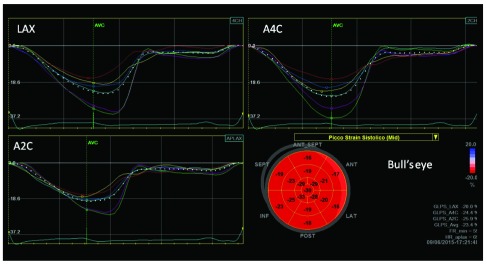
Automated function imaging (AFI) of left ventricular 2-D strain of endurance athlete, showing normal longitudinal regional myocardial deformation despite left ventricular hypertrophy (arrows) (LAX: long-axis view; A4C: apical four- and A2C apical two-chamber views). Bull’s eye represents in a single image all myocardial regional deformations, from basal, to middle and apical segments.

**Figure 3.**
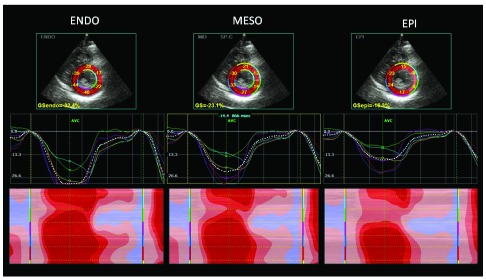
Short-axis left ventricular 2-D strain of endurance athlete, showing optimal regional myocardial deformation of all myocardial layers (arrows) (Epi: epicardium; meso: mesocardium; endo: endocardium).

The athlete’s heart can be considered an interesting model of strain variation at different loading conditions, because there is a LV adaptation of at rest and a load dependency of strain measurement. ( Figure 2 and Figure 3).

In particular, in athletes mild impairment of global longitudinal strain (GLS), lower apical radial strain and lower twisting at rest than in sedentary controls have been observed, together with an increase of basal and middle radial and circumferential strain ^16, 17^. Athletes had higher values for transverse, radial, and circumferential strains when compared with HCM ^18^.

While conventional echocardiographic parameters often failed to distinguish between endurance (runners) and strength (bodybuilders) athlete’s heart, a speckle tracking echocardiography (STE) analysis showed a different pattern of myocardial deformation in these two groups: while global radial strain (GRS) was similar, GLS was lower in runners and global circumferential strain (GCS) was lower in bodybuilders: correlations were found in runners between GLS and end-diastolic volume (r = 0.46; p < 0.05) and body surface area (r = 0.49; p < 0.05), while in bodybuilders, GCS was closely related to LV mass (r = 0.61; p < 0.01) and systolic blood pressure (r = 0.42; p < 0.05) ^19^.

Another study used strain rate imaging to distinguish between individuals with hypertensive LVH and those with strength-training athletic LVH, reporting a significant reduction of systolic and diastolic strain and strain rate in hypertensive individuals, but not in athletes: an e′/a′ ratio >1 was found in 100% of a large population of competitive athletes, 90% of subjects had e′ ≥16 cm/s, s′ ≥10 cm/s, and GLS ≤21% ^20^. Moreover, hearts of hypertensive are characterized by reduced GLS, whereas GCS, GRS, and torsion are similar to those of athletes' hearts: the extent of GLS is strongly associated with LV diastolic function, independently of afterload changes and the degree of LVH ^21^.

Santoro *et al.* stated that LV apical circumferential strain in endurance athletes group was lower than the strength group and control groups (-21.6 ± 4.1% vs. -26.8 ± 7.7%, p < 0.05; vs. -27.8 ± 5.6%, p < 0.01). The endurance group had lower LV twisting (LVT) and untwisting (UTW) than strength group (6.2 ± 0.1° vs. 12.0 ± 0.1°, p < 0.01; -67.3 ± 22.9°/s vs. -122.5 ± 52.8°/s, p < 0.01) and control group (10.0 ± 0.1°, p < 0.01; -103.3 ± 29.3°/s, p < 0.01) ^22^.

Finally, STE showed reduction of longitudinal, circumferential and radial strains and also reduction and delay of peak twisting in triathletes soon after ultralong-duration exercises ^23^.

Three-dimensional (3D) echocardiography offers the ability to improve the diagnostic capability of cardiac ultrasound for evaluating cardiac anatomy, ventricular function, valvular disease and blood flow velocity. This technique is able to quantify LV volume and mass in a fashion which is similar to cardiac magnetic resonance. However, 3D echocardiography is more reproducible, has lower costs and is applicable to a large population of athletes. 3D echocardiography gives more detailed information than two dimensional (2D) echo techniques, providing data on LV remodelling and function; 3D is better in describing morphological features, showing differences in the length and shape of the LV chamber, which are not adequately assessed using 2D technique ^24^.

Using 3D echocardiography, Caselli *et al.* showed LV end-diastolic volumes and mass increased in athletes compared to untrained controls; gender and type of sport had the highest impact on LV remodelling. In particular, male gender and endurance disciplines had the highest impacts on LV end-diastolic volume and mass. Body surface area (BSA) was also an important factor on LV remodelling, while age and blood pressure had only minimal effects. Preserved LV systolic function was observed in athletes, with average values similar in athletes and untrained controls ^25^.

De Castro *et al.* measured LV remodeling index (LVRI) to describe the pattern of LV remodelling in athletes: athletes' LVRI was similar to that of controls, suggesting that the LV remodeling associated with intensive athletic conditioning does not alter LV geometry. Athlete’s heart has a “symmetric” remodeling pattern, because an increased cavity dimension and volume are accompanied by an increased thickness and mass of the ventricles, in the absence LV systolic dysfunction ^26^.

Moreover, isometric activity in strength sports had the highest effects on LV wall thickness, while isotonic activities as marathons had the highest impact on LV diastolic cavity diameter ^27^. The athlete’s heart is therefore characterized by harmonic LV remodelling, differently from patients with hypertrophic or dilated cardiomyopathy ^28^ ( Table 1).

**Table 1.** Athlete’s left heart functional parameters by new echo technologies.

Authors	Journal	Number of Athletes	Type of Sport	Parameter	Mean value	Upper limit
D’Andrea A. *et al.*	J Am Soc Echocardiogr 2010;23:1281–8	650	Endurance/ Power	IVS Tissue Doppler Sm (cm/sec)	13	18
				IVS Tissue Doppler Em (cm/sec)	24	21
				LV Tissue Doppler Sm (cm/sec)	15	20
				LV Tissue Doppler Em (cm/sec)	16	22
				LV Tissue Doppler Em/Am (cm/sec)	1.45	1.7
D’Andrea A. *et al.*	Br J Sport Med 2006;40:244–50	155	Power	LV Intra-ventricular delay (mesc)	9.5	45
Palka P. *et al.*	J Am Coll Cardiol 1997;30:760–8	158	Power	LV myocardial velocity gradient (sec ^-1^)	4.6	7
D’Andrea A. *et al.*	J Am Soc Echocardiogr 2010;23:1281–8	650	Endurance/ Power	LV systolic global longitudinal strain (%)	-17.5	- 22
D’Andrea A. *et al.*	Br J Sports Med. 2008;42(8):696–702	80	Power	LA strain (%)	50	80

### Left atrial function

Atrial function may represent an essential part of cardiac function that is sometimes neglected.

D’Andrea *et al.* investigated whether mechanical dysfunction in the left atrium (LA) is present in patients with either physiological or pathological LVH using two-dimensional strain rate imaging: LA maximum volume was increased but similar between the two groups of patients with LVH. Peak systolic myocardial atrial strain was significantly impaired in patients with pathological LVH compared with controls and athletes. As assessed by multivariate analysis, LV end-diastolic volume/BSA and LV mass in athletes were the only independent factors influencing LA lateral wall peak systolic strain. In contrast, in hypertensive patients, an independent negative association of LA lateral wall peak systolic strain with both LV mass and circumferential end-systolic stress was observed. Moreover, in the overall population of patients with LVH, LA lateral wall systolic strain was an independent predictor of maximum workload during exercise testing ^29^.

## Athlete’s right heart

### Standard echocardiographic analysis

In the recent years, the substantial structural and functional adaptations of the right heart (RH) have been documented, highlighting the complex interplay with the left heart. There is also evolving evidence of acute and chronic cardiac damage, mainly involving the right heart and which may predispose to atrial and ventricular arrhythmias, configuring an exercise-induced cardiomyopathy. Endurance exercise seems to be associated with the greatest extent of cardiac remodelling, involving both LV and right ventricle (RV), while strength training seems to impact minimally on the RV ^30– 33^. Moreover, the reversibility of the changes induced by sport after detraining was considered a typical feature of the athlete’s heart, but several studies have showed that structural and functional recovery might be incomplete, in particular for RV changes and this is particularly true in more practiced athletes ^33^.

Standard echocardiography is the first line imaging exam to differentiate athlete’s heart RV remodeling from pathological conditions. The RH clearly participates in the process of enlargement of the athlete’s heart, with an increase in internal diameters and thickness of its free walls. RV shows greater inflow and outflow dimensions in athletes compared with sedentary controls, with no significant difference in the systolic function. D’Andrea *et al.* documented that RH measures were all significantly greater in highly-trained endurance athletes, compared to age and sex matched strength-trained athletes ^34, 35^.

Typical RV characteristics of the athlete’s heart can resemble those found in arrhythmogenic right ventricle cardiomyopathy (ARVC): in ARVC the enlargement of the RV cavity involves both RV inflow and outflow, and may be associated with RV wall segmental morphological and functional abnormalities; in athletes RV enlargement involves only the inflow tract and systolic function is typically normal ^36^. In addition, the inferior vena cava appeared to be dilated in a study involving 58 endurance athletes ^37^.

LV stroke volume and pulmonary artery systolic pressure (PASP) were found to be powerful independent predictors of both RV and right atrial (RA) dimensions.

Interesting changes in the pulmonary vascular haemodynamics of highly trained athletes can be detected at rest. Concerning the PASP values, whose upper limit of normal was 40 mmHg, endurance-trained athletes showed the highest values, compared with strength trained athletes, and LV stroke volume was an independent predictor of PASP ^38– 40^.

Resting RV global systolic function as measured by fractional area change (FAC) and Tricuspid Annular Plane Systolic Excursion TAPSE seems to be lower in endurance athletes comparing with non-athletic controls. The reduction was more pronounced in the presence of higher RV dilation.

## New right ventricular echocardiographic techniques

Concerning the advanced ultrasound technologies, TDI velocity measurements showed that the early-diastolic phase of LV filling was increased, along with a prolonged isometric relaxation time. LV stroke volume was an independent predictor of the early diastolic velocity (Em) and the time of regional isovolumic release (RTm) of RV free walls ^34^.

As for RV systolic function, both TDI and 2D-strain-derived deformation indexes are reduced at rest in endurance athletes at the RV inlet and mid-free wall level. These changes in RV function at rest are not caused by myocardial damage, in fact there are no increases in NT-proBNP levels among athletes ^41, 42^.

Galderisi *et al.* showed that by combining 3D echo and STE, RV preload exerts its maximal influence on lateral longitudinal fibres (RV lateral longitudinal strain) ^41^.

**Figure 4.**
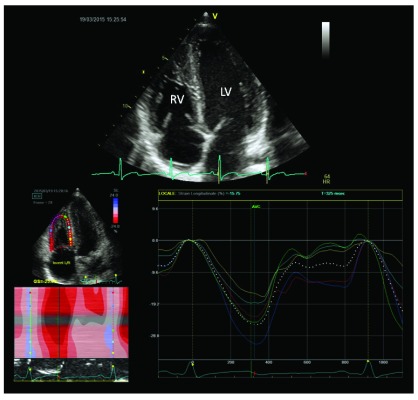
Right ventricular 2-D strain of endurance athlete, showing normal myocardial longitudinal deformation (arrow).

A recent study by D’Andrea *et al.* found comparable 2D and 3D RV systolic indexes between endurance athletes and controls. In this setting, a mild reduction in global RV function could be considered a physiological consequence of RV dilation, since an efficient stroke volume will be reached with higher end-diastolic volumes and then at lower ejection fraction. On the other hand, a severe reduction in RV global systolic function should be considered an abnormal finding even among athletes ^43^ ( Figure 4).

During exercise, increases in both pressures and volumes were greater for the RV, while increases in wall thickness were relatively less than for the LV. As a result, RV wall stress estimates increased 125% during exercise as compared with a modest 14% increase in LV wall stress ^44^.

However, echocardiographic estimates of contractility seem to increase proportional to increases in pulmonary artery pressures during intense exercise of short duration ^45^, suggesting that the RV has the contractile reserve to meet exercise demands, at least for a while.

The RV is more susceptible than the LV to prolonged exercises and is able to induce cardiac fatigue: many studies reported RV dysfunction after long term exercises, as marathons ^46– 50^. D’Andrea *et al.* observed RV dilatation following an ultra-endurance triathlon without changes of LV dimension, by using M-mode, 2D echo and STE (reduction of longitudinal strain about 15% relative to baseline values) ^51^ ( Table 2).

**Table 2.** Athlete’s right heart functional parameters by new echo technologies.

Authors	Journal	Number of Athletes	Type of Sport	Parameter	Mean value	Upper limit
Oxborough D. *et al.*	J Am Soc Echocardiogr 2012;25:263–71	102	Endurance	RV Tissue Doppler Sm (cm/sec)	11	14
				RV Tissue Doppler Em (cm/sec)	10	17
				RV longitudinal strain (%)	-27	-41

## Conclusions

In the last few years, clinical exercise practice, both for recreational and competitive purposes has been spreading worldwide and an increase in the number of subjects with features of exercise-induced cardiac remodeling can be expected. It is important to distinguish healthy, physiological modifications of the athlete’s heart from pathological conditions such as cardiomyopathies.

Cardiac imaging is essential in identifying cardiovascular disease in athletes, but it must be integrated with medical history, symptoms, age, gender, ECG and genetic analyses.

Standard echocardiography has a pivotal role in assessing the athlete’s heart characteristics while the latest developments in ultrasound techniques, such as TDI, 2D strain imaging and 3D echocardiography are important to improve knowledge about physiological and pathological heart remodeling related to sport exercise.
