# Supplementary material for: The role of new echocardiographic techniques in athlete’s heart
Source: F1000Res. 2015 Jul 20;4:289. [Version 1] doi: 10.12688/f1000research.6745.1 (PMC4654447; doi:10.12688/f1000research.6745.1)
Supplement: Peer review history

# Peer review: Referee response for version 1

**DOI:** 10.5256/f1000research.7244.r10561

**Published:** 2015-10-05

**Authors:** Pankaj Garg

**Affiliations:** 1 Leeds Institute of Cardiovascular and Metabolic Medicine, University of Leeds, Leeds, UK

This is a very well structured contemporary review paper on role of echocardiography in subjects with athletic heart. 

Overall, this will add value to this journal. 

Minor suggestions: 

1. In the paragraph of LA function, the introduction line needs a reference. I suggest the following: Leischik *et al. *(2015).
2. Please clearly define that strain will add incremental value to current routine assessment with TDI in finding out individuals who may develop adverse myocardial fibrosis. Add the following references: Hoffman *et al.*(2014), O'Keefe *et al.* (2012).

I have read this submission. I believe that I have an appropriate level of expertise to confirm that it is of an acceptable scientific standard.

# Peer review: Referee response for version 1

**DOI:** 10.5256/f1000research.7244.r9757

**Published:** 2015-08-03

**Authors:** Jarosław Kasprzak

**Affiliations:** 1 Department of Cardiology, Medical University of Łódź, Łódź, Poland

The paper is a current, high quality review of the novel echocardiographic approaches to quantification of myocardial function in the context of training-induced remodeling.

The authors provide a fair overview of methods used for discriminating right and left ventricular pathology which is important for sudden death risk stratification in this subset.

The review is of interest for the readership and I suggest to accept it on "as is" basis.

I have read this submission. I believe that I have an appropriate level of expertise to confirm that it is of an acceptable scientific standard.
